# Ketamine Use in a Laboring Mother With Type 4 Ehlers-Danlos Syndrome, a Family History of Malignant Hyperthermia, and Opioid-Induced Seizures

**DOI:** 10.7759/cureus.87471

**Published:** 2025-07-07

**Authors:** Ryan I Bower, Ian W McArdle, Arpan Kohli, Merv Unger

**Affiliations:** 1 Anesthesiology and Perioperative Medicine, West Virginia University School of Medicine, Morgantown, USA

**Keywords:** ehlers-danlos syndrome type 4, high-risk labor delivery, ketamine during labor, labor analgesia, obstetric anesthesia

## Abstract

Ehlers-Danlos Syndrome (EDS) is a group of inherited, autosomal dominant connective tissue disorders caused by defects in procollagen and collagen production. Vascular EDS, also known as type 4 EDS, involves mutations in the COL3A1 gene, resulting in fragile vascular tissues and compromised organ integrity, which pose significant challenges during both labor and anesthesia. We report the case of a 19-year-old female patient with type 4 EDS, opioid-induced seizures, and a family history of malignant hyperthermia, who presented to the labor ward at 37 weeks of gestation. Due to her complex medical history, traditional methods for labor analgesia, including neuraxial techniques and remifentanil patient-controlled analgesia, were associated with significantly increased risks. A tailored pain management regimen was implemented, consisting of intravenous acetaminophen, cyclobenzaprine, and a ketamine infusion. The patient delivered a healthy neonate without complications. This case highlights the successful use of ketamine for labor analgesia in a high-risk obstetric patient with EDS.

## Introduction

Ehlers-Danlos Syndrome (EDS) is a group of 13 inherited connective tissue disorders caused by defects in procollagen and collagen production [1,2]. Vascular EDS, also known as Type IV EDS, results from a defect in the COL3A1 gene, which encodes the alpha-1 chain of type III collagen [1]. This mutation leads to fragility of vascular tissues and hollow organs [1]. Type 4 EDS can be diagnosed using the Villefranche criteria, which include easy bruising, excessive bleeding, arterial, intestinal, or uterine fragility, thin or translucent skin, and other minor criteria [1,3]. Clinical suspicion is confirmed by fibroblast culture showing defective collagen or genetic testing revealing a COL3A1 mutation. These manifestations require special consideration during anesthesia and other interventions, particularly with neuraxial anesthesia, central vascular access, hemodynamic management, and fluid resuscitation. Although neuraxial anesthesia has been performed without difficulty in some cases, there remains a theoretical increased risk of epidural hematoma [4,5]. Dolan et al. recommend avoiding neuraxial anesthesia entirely in these patients based on a review of two case reports [4]. One report described intra-abdominal hemorrhage resulting in death following exploratory laparotomy in a patient with type IV EDS who had multiple related complications, including severe epistaxis, menorrhagia, melena, and spontaneous hemorrhage from a retro-orbital aneurysm. This patient had delivered a newborn just one week prior and developed a spontaneous perineal hematoma during the second stage of labor, requiring multiple transfusions. The second case report described a patient who developed large hematomas in multiple extremities after warfarin administration for deep vein thrombosis and pulmonary embolism, further illustrating the bleeding risk in type IV EDS [4]. These cases underscore the increased risk of hemorrhage and morbidity in this population. By extension, the same bleeding risk could apply to epidural placement, making epidural hematoma a concerning possibility. Although most women with Type IV EDS undergo pregnancy and delivery without complications, literature on obstetric anesthesia care for this condition remains scarce [4]. Additional risks during labor include uterine rupture, poor wound healing, and failed hemostasis following cesarean delivery [6]. Maternal mortality rates of up to 25% have been reported in laboring patients with vascular EDS [6]. Given these potentially life-threatening risks, every aspect of care must be carefully considered and planned. Further case reports and research are needed to guide anesthetic management in this patient population.

In anesthetic practice, few established guidelines exist for laboring patients with Type IV EDS, as all delivery and analgesia options carry increased risks. Vaginal delivery poses the threat of uterine or arterial rupture, particularly during the second stage of labor [2], while cesarean section increases the risk of intraoperative bleeding and postoperative wound complications. Although combined spinal-epidural anesthesia has been performed safely in some cases, the theoretical risk of epidural hematoma persists in both vaginal and cesarean deliveries [2,7]. A remifentanil patient-controlled analgesia (PCA) pump may be considered as an alternative analgesic strategy; however, this was contraindicated in our patient due to a history of opioid-induced seizures. This case report presents a unique labor analgesia strategy in a patient with type IV EDS, a history of opioid-induced seizures, and a family history of malignant hyperthermia.

## Case presentation

This case involves a 19-year-old female, G1P0, with a history of morbid obesity (BMI 41.9), type 4 EDS, scoliosis, PTSD, headaches, fibromyalgia, anxiety, depression, and gestational hypertension. She presented at 37 weeks and 3 days of gestation with labor contractions. Her admission blood pressure was 139/82 mmHg. The patient’s allergy list included a childhood history of opioid-induced seizures. Her mother had been advised that the patient should never receive opioids, “including remifentanil.” Since that time, she had not been administered opioids per this recommendation. Her family history was notable for malignant hyperthermia. Given her type 4 EDS diagnosis, a transthoracic echocardiogram (TTE) was performed and showed normal biventricular function without aortic dilation. Twenty-four hours after admission, her contractions became increasingly uncomfortable. During anesthesia evaluation, she was classified as American Society of Anesthesiology Physical Status III. Admission platelet count was within normal limits. Neuraxial anesthesia was avoided due to the increased risk of epidural hematoma, and opioid analgesics, including remifentanil, were contraindicated due to a prior history of opioid-induced seizures. Following shared decision-making with the patient regarding epidural placement, remifentanil PCA, and adjunct treatment, the initial anesthetic plan included intravenous acetaminophen and a pudendal nerve block. Ketamine was discussed as an alternative option, including counseling on the potential for dissociative hallucinations. The patient was also informed that ketamine has minimal effects on the fetus. However, due to a preference to avoid the augmented sensory experience of labor associated with ketamine, the patient initially declined ketamine analgesia.

**Figure 1:**
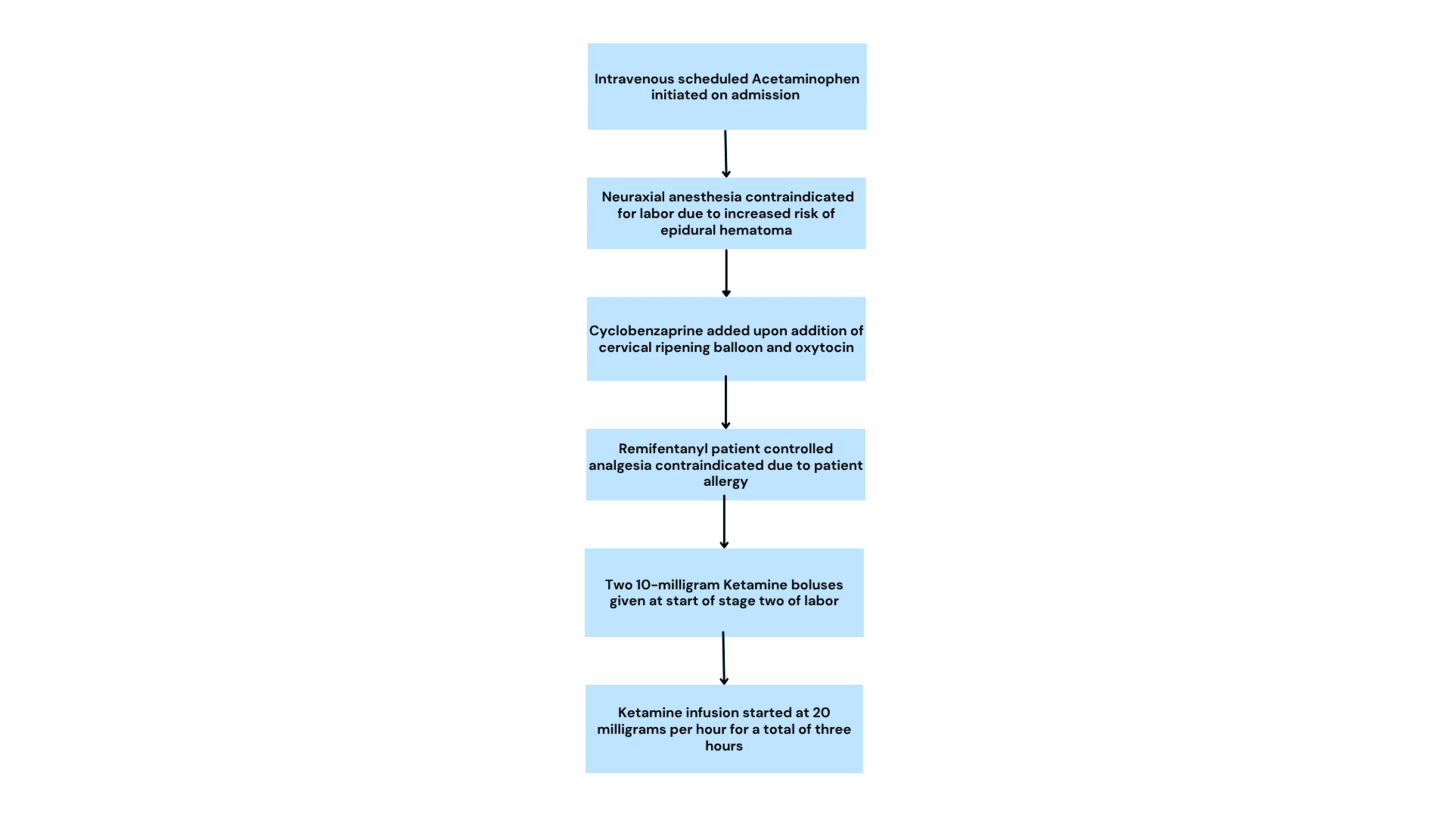
Analgesia plan for patients with vascular subtype Ehlers-Danlos syndrome

The patient’s labor progressed over the next 24 hours with the administration of cervical misoprostol, a cervical ripening balloon, and oxytocin. Cyclobenzaprine was added as part of a multimodal analgesia regimen. After 48 hours, the patient entered the second stage of labor, and both ketamine and a pudendal nerve block were again offered due to uncontrollable pain. At this point, the patient declined the pudendal nerve block but consented to ketamine administration. She received two 10 mg ketamine boluses, spaced 20 minutes apart, followed by a continuous infusion at 20 mg per hour. Active labor lasted approximately two hours, culminating in the delivery of a female neonate with APGAR scores of 8 and 9 at one and five minutes, respectively. While pain scores were not formally documented during labor, the patient reported substantial pain relief following ketamine initiation. The neonate remained vitally stable and stayed with the mother after birth. Fetal heart rate was within acceptable limits throughout delivery. The patient sustained first-degree perineal and peri-clitoral lacerations, both of which were repaired. The ketamine infusion was continued for one additional hour post-delivery, for a total of three hours of administration, including during the repair of the lacerations. The full progression of the analgesic plan is summarized in Figure 1. The patient’s blood pressure remained stable throughout, with a maximum of 147/75 mmHg during ketamine administration. She remained in the postpartum unit for 24 hours, with adequate pain control and no additional analgesia required. The patient was discharged without complication.

## Discussion

EDS is a family of connective tissue disorders that impair collagen formation throughout the body, resulting in joint laxity, easy bleeding/bruising, vascular fragility, smooth muscle weakness, and poor connective tissue repair [1,2]. Type 4 EDS poses significant risks for patients undergoing anesthetic or surgical procedures. These include an increased likelihood of hemorrhage, hematoma formation, hollow organ rupture, and poor incisional healing. We presented the case of a 19-year-old parturient requiring a uniquely tailored anesthetic plan due to her type 4 EDS, contraindication to opioids, and family history of malignant hyperthermia, each of which complicates traditional labor anesthesia strategies. Given her diagnosis and clinical background, vaginal delivery was favored over cesarean section to reduce the risk of catastrophic hemorrhage, impaired wound healing, secondary infection, and rupture of adjacent hollow organs. While epidural anesthesia is the typical choice for labor analgesia, it was contraindicated in this patient due to the increased risk of epidural hematoma associated with type 4 EDS. Similarly, a cesarean section under neuraxial anesthesia carried comparable risks. Moreover, should an emergent cesarean delivery become necessary, the use of volatile anesthetic agents and succinylcholine would be contraindicated due to the family history of malignant hyperthermia. Additionally, adequate analgesia posed a challenge, as opioids, including remifentanil, were contraindicated due to the patient’s history of opioid-induced seizures. Although remifentanil has been included in multimodal analgesic plans for patients with type 4 EDS, it was not a safe option in this case [2]. Taking all clinical factors into account, a plan was developed involving acetaminophen, cyclobenzaprine, and a ketamine infusion with boluses, along with a strategy for vaginal delivery after thorough shared decision-making with the patient. If emergent cesarean section had been required, the safest approach would have involved total intravenous anesthesia (TIVA) with rocuronium for neuromuscular blockade, and adjunct agents such as ketamine, nitrous oxide, and dexmedetomidine to achieve adequate analgesia and anesthesia.

Minimal literature exists regarding ketamine use in laboring patients, and there are currently no documented reports of ketamine use in a parturient with type 4 EDS. A previous randomized controlled trial comparing low-dose ketamine (0.2 mg/kg loading dose and 0.2 mg/kg/hour infusion rate) to placebo demonstrated a significant reduction in perceived labor pain in otherwise healthy parturients [8]. Another study of 73 women found that 85% of those who received ketamine infusion during vaginal delivery reported “relief from labor pain,” with no significant maternal or fetal side effects [9]. These findings suggest that ketamine can provide adequate labor analgesia in select patients. Although pain scores were not formally recorded during active labor in our case, the patient subjectively reported improved pain control after ketamine administration. Given the known collagen defect in type 4 EDS, our patient was at increased risk for large vessel rupture, particularly during periods of hemodynamic stress such as labor. Ketamine’s indirect sympathomimetic effects posed a potential concern, as they could exacerbate hemodynamic fluctuations. Therefore, balancing ketamine’s analgesic benefits with its cardiovascular effects was critical in choosing it as an analgesic agent. However, the patient’s normal TTE on admission lowered the suspicion for underlying aortic pathology, such as dissection or aneurysm. It was reasoned that a conservative dosing strategy (a 10 mg loading dose administered twice, 20 minutes apart, followed by a continuous infusion at 20 mg/hour) could reduce the risk of sudden blood pressure elevations while still providing adequate analgesia. Upon review, the patient’s pain was well controlled, and she experienced minimal side effects, supporting the conclusion that ketamine was an effective and appropriate analgesic in this complex case. Blood pressure remained largely stable, with frequent monitoring every five minutes during labor as per the institution’s standard delivery protocol.

Moreover, concerns regarding ketamine’s effects on neonatal function following maternal exposure were carefully considered. Ellingson et al. demonstrated that ketamine readily crosses the placental barrier in laboring patients, producing transient but significant hypertension only in the mother [10]. The theoretical risks of increased sympathetic tone and excessive secretions raised concerns about the potential to induce or exacerbate neonatal respiratory distress. However, studies by Joel et al. and Havle et al. found that among a total of 145 neonates exposed to maternal ketamine during labor, all had APGAR scores greater than 4, suggesting a low risk of adverse neonatal outcomes [8,9]. In our case, the neonate had APGAR scores of 8 and 9 at one and five minutes, respectively. Neither the parturient nor the neonate experienced any side effects associated with ketamine administration, further supporting its potential role as a safe alternative for labor analgesia augmentation in complex obstetric cases.

## Conclusions

The use of ketamine as the primary analgesic proved to be a successful alternative for vaginal delivery in a patient with type 4 EDS, contraindications to opioids, and a family history of malignant hyperthermia. Conventional labor analgesia options were contraindicated due to the patient’s complex medical history. Despite these limitations, the patient experienced an uncomplicated vaginal delivery, reported minimal pain during the second stage of labor, and delivered a healthy neonate with no observable adverse effects. This case demonstrates that ketamine can provide safe and effective analgesia in laboring patients with high-risk conditions such as type 4 EDS, offering a viable alternative when standard analgesic approaches are not suitable.
